# The co-chaperone Cdc37 regulates the rabies virus phosphoprotein stability by targeting to Hsp90AA1 machinery

**DOI:** 10.1038/srep27123

**Published:** 2016-06-02

**Authors:** Yunbin Xu, Fei Liu, Juan Liu, Dandan Wang, Yan Yan, Senlin Ji, Jie Zan, Jiyong Zhou

**Affiliations:** 1 Key Laboratory of Animal Virology of Ministry of Agriculture, Zhejiang University, Hangzhou 310058, P. R. China; 2 Collaborative Innovation Center and State Key Laboratory for Diagnosis and Treatment of Infectious Diseases, First Affiliated Hospital, Zhejiang University, Hangzhou 310003, P. R. China; 3 College of Veterinary Medicine, Nanjing Agricultural University, Nanjing 210095, P. R. China

## Abstract

Cdc37, as a kinase-specific co-chaperone of the chaperone Hsp90AA1 (Hsp90), actively aids with the maturation, stabilization and activation of the cellular or viral kinase/kinase-like targets. Phosphoprotein (P) of rabies virus (RABV) is a multifunctional, non-kinase protein involved in interferon antagonism, viral transcription and replication. Here, we demonstrated that the RABV non-kinase P is chaperoned by Cdc37 and Hsp90 during infection. We found that Cdc37 and Hsp90 affect the RABV life cycle directly. Activity inhibition and knockdown of Cdc37 and Hsp90 increased the instability of the viral P protein. Overexpression of Cdc37 and Hsp90 maintained P’s stability but did not increase the yield of infectious RABV virions. We further demonstrated that the non-enzymatic polymerase cofactor P protein of all the genotypes of lyssaviruses is a target of the Cdc37/Hsp90 complex. Cdc37, phosphorylated or unphosphorylated on Ser13, aids the P protein to load onto the Hsp90 machinery, with or without Cdc37 binding to Hsp90. However, the interaction between Cdc37 and Hsp90 appears to have additional allosteric regulation of the conformational switch of Hsp90. Our study highlighted a novel mechanism in which Cdc37/Hsp90 chaperones a non-kinase target, which has significant implications for designing therapeutic targets against Rabies.

Viruses, as obligate intracellular parasites, have evolved to use many host cell proteins to help their efficient replication and spread. Rabies virus (RABV), as a fatal neurotropic virus in humans, is a prototype virus of the Lyssavirus genus belonging to the Rhabdoviridae family^1^,^2^. Its single, negative-stranded RNA genome of 11928~11932 nucleotides is encapsidated by the nucleoprotein (N), which is associated with large (L) polymerase protein and the non-enzymatic polymerase cofactor phosphoprotein (P). The nucleocapsid has a tightly coiled, helical structure that is associated with the matrix protein (M), and surrounded by a membrane containing the glycoprotein (G) and other host cell-derived membrane proteins. After the virus enters the host cell via a low-pH-induced membrane fusion process catalyzed by G, viral transcription and replication processes are then catalyzed by the L-P polymerase complex. During RABV infection, viral transcription and replication are carried out in the intracellular Negri Bodies (NBs), which contain viral proteins and cellular proteins, such as TLR3, Hsp70, Hsp90 and CCTγ^3^,^4^,^5^,^6^. In addition, NBs sequester misfolded proteins or overexpressed proteins when cellular stress occurs^3^,^4^,^7^. Understanding the potential interactions of cellular proteins with these viral proteins involved in the formation of NBs is important to determine the mechanism of RABV infection.

Heat shock protein 90 (Hsp90) is a conserved molecular chaperone that is ubiquitously expressed in eukaryotic cells, playing important roles in the regulation of protein folding, maturation and activation to maintain cellular homeostasis and survival^8^,^9^. The conformation and activity of Hsp90 are regulated by the binding of ATP to its N-terminal binding domain (NBD). Upon ATP binding, the NBD of Hsp90 switches to the “closed” state, allowing Hsp90 to clamp onto the target protein, assisting conformational maturation of the target and maintaining the protein in an active state to exert its function^10^. The ATPase activity of Hsp90 cleaves the ATP into ADP and Pi, leaving Hsp90 in the “open” state and releasing the target protein from Hsp90^11^,^12^. Inhibitors such as geldanamycin and its derivative analog 17-(Allylamino)-17-demethoxygeldanamycin (17-AAG) inhibit the function of Hsp90 by binding to its ATP-binding pocket, thereby locking the conformation of Hsp90 in the “open” state, leading to subsequent target protein misfolding and degradation^13^,^14^,^15^.

Unlike the more general Hsp70 and Hsp60 chaperones, Hsp90, in concert with a defined set of co-chaperones, appears to have substrate specific binding activity. Cdc37 is a highly specialized co-chaperone of Hsp90 that serves as an adaptor to target Hsp90 to a subset of cellular kinases and aids Hsp90 with target stabilization and activation^16^. Cdc37 interacts with the NBD of Hsp90 such that the Hsp90 ATPase cycle is inhibited, thereby permitting the loading of target proteins^17^. Therefore, the interaction of Cdc37 with Hsp90 has long been thought as essential to chaperone target proteins. A Cdc37 mutant defective in Hsp90 binding also functioned in a dominant-negative fashion by preventing the interaction between Hsp90 and kinases^18^,^19^,^20^. Inhibitors, such as celastrol, lead to target degradation by disruption of Cdc37/Hsp90 complexes, without interfering with ATP binding to Hsp90^21^,^22^. Surprisingly, it was shown recently that the binding of Cdc37 with Hsp90 is not required for its stabilization function; however, the activity of Hsp90 is indispensable^23^.

Our recent study showed that the cytoplasmic Hsp90 colocalizes with the viral nucleoprotein (N) and phosphoprotein (P) in NBs of RABV-infected cells^6^. Here, we aimed to understand whether physical colocalization of Hsp90 with RABV viral N and P proteins has any functional relevance. We found that the RABV life cycle correlates directly with the expression of Cdc37 and Hsp90. We further demonstrated that the non-enzymatic polymerase cofactor P protein is a target of the Cdc37/Hsp90 complex. Cdc37 helps the P protein to load onto the Hsp90 machinery, with or without Cdc37 binding to Hsp90. However, unlike the chaperoning of the kinase targets, phosphorylation of Cdc37 is not required for P protein stabilization. Our study highlighted a novel mechanism whereby Cdc37/Hsp90 chaperones a non-kinase target.

## Results

### Rabies virus infection increases the expression of cellular proteins Cdc37 and Hsp90

**Figure 1:**
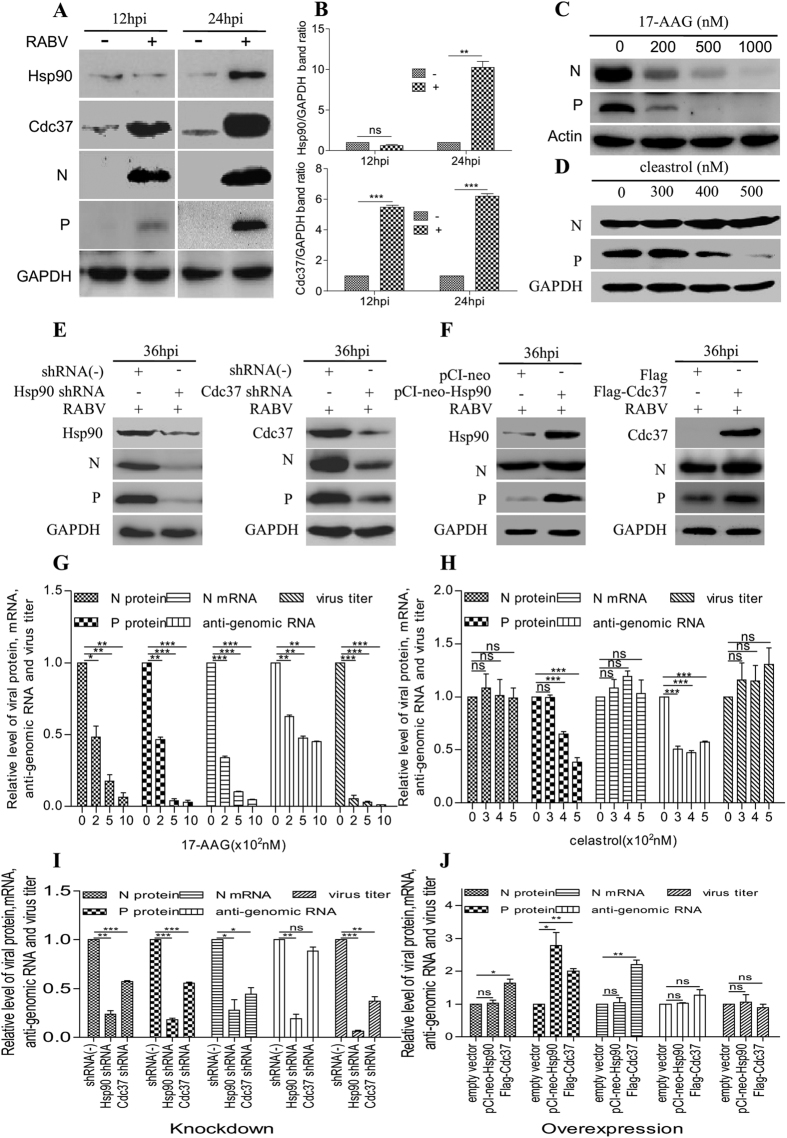
The activities of cellular proteins Cdc37 and Hsp90 are required for RABV infection. (**A**) N2a cells were infected with RABV strain HEP-Flury at an MOI = 1. At 12 hpi and 24 hpi, collected cell lysates were probed with the mouse mAbs to N and P viral proteins, rabbit mAbs to Cdc37 and Hsp90, and rabbit anti-GAPDH pAb in immunoblotting experiments. (**B**) Quantitative analysis of cellular proteins Cdc37 and Hsp90 described in A. (**C**,**D**) N2a cells were incubated with RABV strain HEP-Flury at an MOI = 1 for 2 h, and then treated with 17-AAG for 22 h or with celastrol for 34 h at the indicated doses. Immunoblots of cell lysates were probed with rabbit anti-ß-actin and anti-GAPDH pAbs and mouse mAbs to N and P to detect the expression of N and P respectively. (**E**) N2a cells were transfected with Cdc37 shRNA or Hsp90 shRNA for 24 h, and then infected with RABV strain HEP-Flury at an MOI = 1 for 36 h. The shRNA (−) was used as the negative control. The expressions of N, P, Cdc37 or Hsp90 were determined using mouse mAbs to N and P and rabbit mAbs to Cdc37 and Hsp90 by western blotting. (**F**) N2a cells were transfected with pCI-neo-Hsp90 or pCMV-N-Flag-Cdc37 for 24 h, and then infected with RABV strain HEP-Flury at an MOI = 1 for 36 hpi. The expressions of N, P, Hsp90 or Cdc37 were determined by western blotting. The corresponding empty vector was used as the negative control. (**G**–**J**) Quantitative analysis of viral proteins, viral N mRNA, anti-genomic RNA and virus titer in (**C**–**F**) described experimental samples. Total cellular RNA of the samples described in (**C**–**F**) was analyzed in comparative qRT-PCR. Titers of infectious RABV progeny for samples described in (**C**–**F**) were determined by a standard plaque assay into BHK-21 cells to assay the virus production. Error bars: Mean ± SDs of three independent experiments. ^ns^*P* > 0.05, ^*^P < 0.05, ^**^P < 0.01, ^***^P < 0.001.

Our recent report demonstrated the colocalization of the cytoplasmic chaperone Hsp90 with the hollow ring-like structure of NBs containing viral N and P proteins^6^. To determine the detailed dynamic profile of Hsp90, RABV-infected N2a cells were evaluated at 12 and 24 hours post-infection (hpi) by immunoblotting (Fig. 1A,B). Compared with mock-infected cells, Hsp90 protein expression showed no significant changes in RABV-infected cells at 12 hpi (*P* > 0.05), but increased significantly at 24 hpi (*P* < 0.01). Correspondingly, the accumulation of Cdc37, the Hsp90 co-chaperone, was markedly promoted after RABV infection at both 12 hpi and 24 hpi (*P* < 0.001), before Hsp90 upregulation. These results indicated that RABV infection upregulated the expression of both Cdc37 and Hsp90.

We then analyzed whether chaperone proteins Cdc37 and Hsp90 in turn affected RABV infection. We measured the dynamics of RABV infection under conditions of activity inhibition, knockdown or overexpression of Cdc37 and Hsp90. Firstly, the activity of Hsp90 was inhibited with 17-AAG in RABV infected cells; we found that the level of viral N and P proteins, viral N mRNA, viral anti-genomic RNA and infectious RABV progeny were all significantly reduced, in a dose-dependent manner (Fig. 1C,G, P < 0.05, 0.01 or 0.001), suggesting a positive role of Hsp90 in regulating RABV infection. Secondly, the interaction between Cdc37 and Hsp90 was inhibited by celastrol in RABV infected cells. We observed that the level of the viral P protein was reduced in a dose-dependent manner (Fig. 1D,H, P < 0.001). The level of the viral N protein, viral N mRNA and infectious RABV progeny showed non-significant changes, while viral anti-genomic RNA decreased at all concentrations tested (Fig. 1D,H, P < 0.001). Cell viability of N2a cells did not change significantly with different concentrations of 17-AAG or celastrol (Supplementary Fig. 1A,B). These results indicated that the interaction between Cdc37 and Hsp90 plays an important role in the P protein accumulation and the N protein appeared to be regulated differently from the P protein. Thirdly, to rule out the possibility of spurious off-target or side effects of 17-AAG and celastrol, Cdc37 or Hsp90 was knocked down in RABV infected cells. We found that the level of viral N and P proteins, viral N mRNA, viral anti-genomic RNA and infectious RABV progeny were all significantly reduced, with the exception that viral anti-genomic RNA showed non-significant changes when Cdc37 was knocked down (Fig. 1E,I), suggesting that celastrol has side effects during RABV infection. Finally, Cdc37 or Hsp90 was overexpressed in RABV infected cells. We observed that at 36 hpi, the level of viral N and P proteins were significantly elevated when Cdc37 was overexpressed; however, only the level of the viral P protein increased when Hsp90 was overexpressed. In contrast, both the virus titer and anti-genomic RNA of RABV showed a non-significant change (Fig. 1F,J, P > 0.05). Interestingly, at 36 hpi, the viral mRNA did not change significantly with Hsp90 overexpression, but changed significantly with Cdc37 overexpression. We further shortened the infection time, and found that at 24 hpi, only the level of viral N and P proteins showed significant elevation; viral mRNA did not change significantly with Cdc37 overexpression (Supplementary Fig. 1C,D). These data demonstrated that Cdc37 and Hsp90 overexpression increased the accumulation of viral N and/or P proteins, but did not enhance the production of infectious RABV progeny. Taken together, these data indicated that the cellular proteins Cdc37 and Hsp90 were required during the RABV life cycle, and that Cdc37 and Hsp90 affect directly viral protein accumulation at the protein level, but not at the transcription level during RABV infection.

### Both Cdc37 and Hsp90 promote the stability of viral P protein during RABV infection

**Figure 2:**
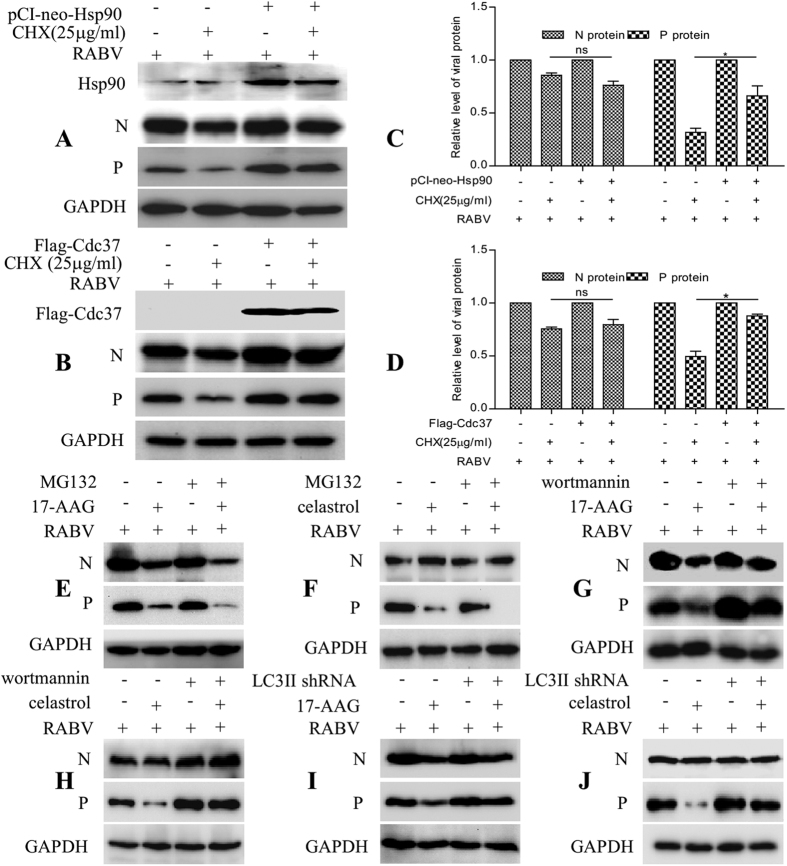
Cdc37 or Hsp90 maintain viral P protein stability by preventing protein degradation. (**A**,**B**) N2a cells were infected with RABV strain HEP-Flury at an MOI = 1 after transfection with pCI-neo-Hsp90 or pCMV-N-Flag-Cdc37 for 24 h, and were cultured for 18 h. At 18 hpi, the resultant cells were treated with CHX (25 μg/ml) for 6 h and then harvested to detect the expression of Cdc37, Hsp90, N and P. Immunoblot analysis was performed using their corresponding antibodies. The empty vector transfected and Cdc37/Hsp90-overexpressing cells without CHX were used as controls. (**C**,**D**) Quantitative analysis of N and P proteins described in A and B. (**E**–**H**) N2a cells were incubated with RABV strain HEP-Flury at an MOI = 1 for 2 h, and were then treated with 17-AAG (500 nM) for 22 h or with celastrol (500 nM) for 34 h. The resultant cells were incubated with MG-132 (10 μM) for 6 h or with wortmannin (500 nM) for 22 h and then harvested for immunoblot analysis of viral proteins N and P. (**I**,**J**) N2a cells were incubated with RABV strain HEP-Flury at an MO1 = 1 for 2 h after transfection with LC3II shRNA or negative control shRNA for 24 h, and then treated with 17-AAG (500 nM) for 22 h or with celastrol (500 nM) for 34 h. The resultant cells were harvested for immunoblot analysis. Immunoblot assays were performed using their corresponding antibodies to detect the expression of viral proteins P and N. Error bars: Mean ± SDs of three independent experiments. ^ns^*P* > 0.05, ^*^P < 0.05, ^**^P < 0.01, ^***^P < 0.001.

To assess whether the cellular proteins Cdc37 and Hsp90 affect the synthesis or stability of viral proteins during RABV infection, Cdc37 or Hsp90 overexpressing cells were infected with RABV and cultured with cycloheximide (CHX; 25 μg/ml), a protein synthesis inhibitor. Western blotting analysis of protein abundance showed that at 6 h after CHX treatment, the viral P protein had a higher concentration; however, the viral N protein showed non-significant changes in Cdc37 or Hsp90 overexpressing cells compared with CHX treated cells without Cdc37 or Hsp90 overexpression (Fig. 2A,B, quantified in Fig. 2C,D, P < 0.05). This data demonstrated that overexpression of Cdc37 and Hsp90 caused the accumulation of viral P proteins, even under decreasing protein synthesis. This result suggested that the stability of the viral P protein was maintained by Cdc37 and Hsp90.

To validate this hypothesis, RABV-infected N2a cells were treated with MG-132, a proteasome inhibitor, in the presence of 17-AAG or celastrol. The results indicated that in 17-AAG-treated cells, the viral N and P proteins were present in lower concentrations than in untreated cells (Fig. 2E, Supplementary Fig. 2A, P < 0.05). Further treatment with MG-132 in the presence of 17-AAG did not restore, but further decreased the relative level of viral P and N proteins significantly (Fig. 2E, Supplementary Fig. 2A, P < 0.05). In celastrol-treated cells, the viral P protein showed similar changes to that in 17-AAG-treated cells, with or without MG-132, while the viral N protein was not affected (Fig. 2F, Supplementary Fig. 2B, P < 0.05). To demonstrate that MG-132 is working in all the experiments, we used Cdc2 as a control, which is a known substrate of Hsp90^24^. The effect of 17-AAG on Cdc2 degraded via the proteasome pathway^25^, which was reduced by MG-132 (Supplementary Fig. 2G), indicating that MG-132 used in our research is working. This data indicated that the viral P protein is unstable when the activities of Cdc37 and Hsp90 are inhibited, and that the viral N protein is unstable when the activity of Hsp90 is inhibited, irrespective of proteasome activity.

Interestingly, in the presence of 17-AAG combined with wortmannin, an autophagy inhibitor, the expression levels of the viral P and N proteins, which were originally reduced due to 17-AAG inhibition, were restored in RABV-infected cells (Fig. 2G, Supplementary Fig. 2C, P < 0.01 or < 0.001). In the presence of celastrol, treatment with wortmannin also restored the expression level of the viral P protein; however, but celastrol or wortmannin treatment did not affect the viral N protein significantly (Fig. 2H, Supplementary Fig. 2D, P < 0.05). Meanwhile, the level of IκB kinase-α (IKKα), an established client protein of Hsp90 degraded via the autophagy pathway^25^,^26^, was reduced after treatment with 17-AAG, and restored by wortmannin (Supplementary Fig. 2H), indicating that wortmannin used in our research is also working. Furthermore, an LC3 II, a key autophagy pathway component, short hairpin RNA (shRNA) experiment also showed that the viral P and N proteins in 17-AAG-treated cells with LC3 II knockdown had a slower degradation than that in 17-AAG treated cells without LC3 II knockdown after RABV infection. The viral P protein, but not the viral N protein, in celastrol treated cells with LC3 II knockdown, showed slower degradation than that in celastrol only-treated cells without LC3 II knockdown after RABV infection (Fig. 2I,J, Supplementary Fig. 2E,F,I, P < 0.05 or < 0.01). These findings were consistent with Fig. 2A,B, and further demonstrated that the chaperone proteins Cdc37 and Hsp90 improved the stability of the viral P protein, and Hsp90 improved the stability of the viral N protein for RABV infection by preventing it from clearance through autophagy.

### The viral P protein, as a target protein, interacts with cellular proteins Cdc37 and Hsp90

**Figure 3:**
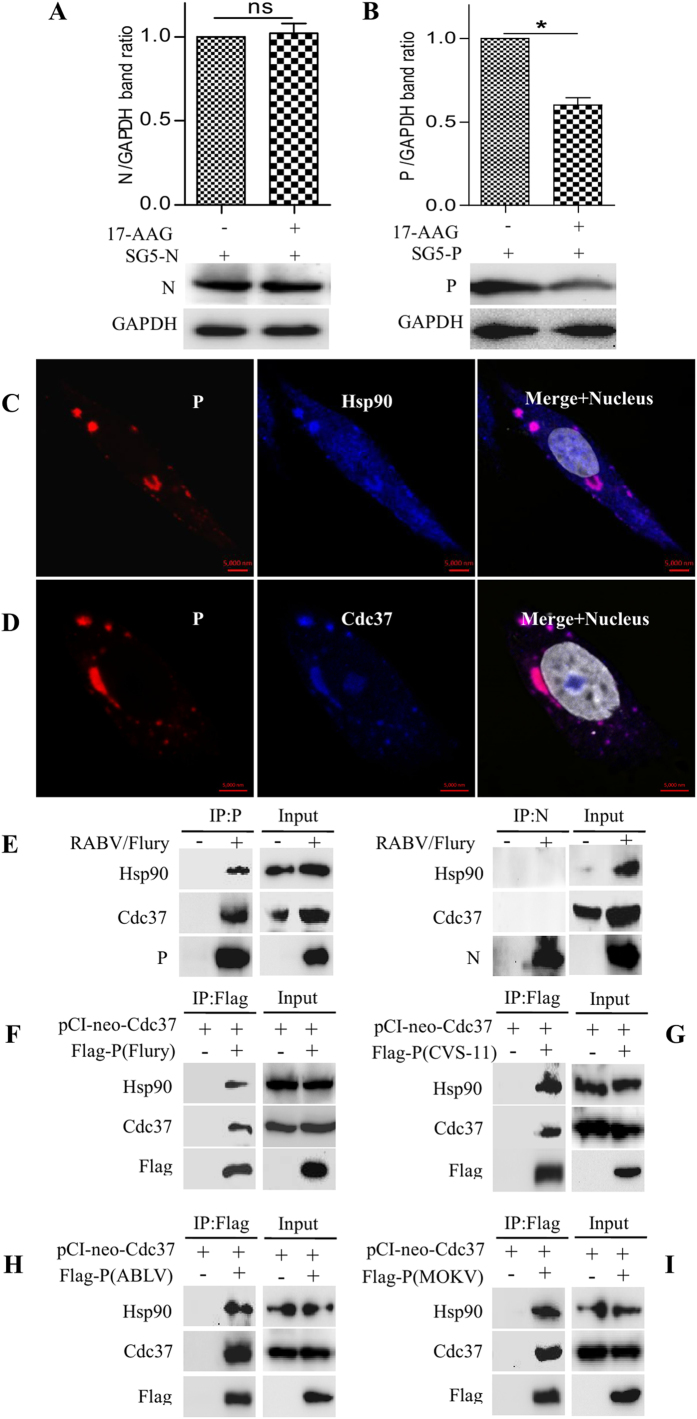
Cellular chaperone Hsp90 and its co-chaperone Cdc37 affect the stability of viral P protein by protein-protein interaction. (**A**,**B**) N2a cells were transfected with pSG5-N or pSG5-P, and 6 h later were incubated with 17-AAG (250 nM) for 42 h. The stabilities of viral N and P proteins were determined in the transfected cells by immunoblotting using their antibodies. Error bars: Mean ± SDs of three independent experiments. ^ns^*P* > 0.05, ^*^P < 0.05. (**C**,**D**) N2a cells were infected with RABV strain HEP-Flury for 24 h. The resultant cells were stained with mouse anti-P mAb, rabbit mAbs to Cdc37 and Hsp90, and observed under confocal microscopy. (**E**) The total cell lysates from RABV strain HEP-Flury infected N2a cells were immunoprecipitated with mouse mAb to viral protein P or N. Finally, the Cdc37, Hsp90, viral proteins P and N in the immune complex were analyzed by western blotting. (**F**–**I**) N2a cells were co-transfected with pCI-neo-Cdc37 together with Flag-P (Flury), Flag-P (CVS-11), Flag-P (ABLV) and Flag-P (MOKV) respectively. Protein extracts were immunoprecipitated with mouse anti-Flag mAb and immunoblotted with anti-Flag, and rabbit mAbs to Cdc37 and Hsp90.

To further analyze whether the viral P or N protein is a target protein of Hsp90, we constructed the recombinant vectors pSG5 containing the P or N gene and transfected these constructs into N2a cells. When these transfected cells were treated with 17-AAG, viral P protein, but not N, was markedly degraded (Fig. 3A,B, P < 0.05), suggesting that the P protein, but not the N protein is the target protein of Hsp90.

We then investigated whether the maintenance of the stability of the viral P protein involves binding to Hsp90 and to its co-chaperone Cdc37. First, the colocalization of the viral P protein with Cdc37 and Hsp90 was analyzed in the RABV infected N2a cells and in N2a cells transfected with the individual P protein from plasmid. The confocal image showed that the P protein colocalized with Cdc37 and Hsp90 (Fig. 3C,D, Supplementary Fig. 3A,B). Subsequently, a co-immunoprecipitation assay (Co-IP) was performed with RABV infected cells to further analyze whether the colocalization involves protein-protein interactions. The Co-IP data demonstrated that the endogenous proteins Cdc37 and Hsp90 could be immunoprecipitated with mouse anti-P mAb, but not with anti-N mAb in infected cells (Fig. 3E), although the colocalization of N with Cdc37 and Hsp90 was observed (Supplementary Fig. 3C,D). To further validate this interaction and to eliminate disturbance of other viral protein encoded by RABV, N2a cells co-transfected with the plasmids Flag-tagged P and PCI-neo-Cdc37 were detected with anti-Flag antibody as bait for Cdc37/Hsp90 complex. We found that the Cdc37/Hsp90 complex could bind P (Fig. 3F). These data showed that the viral P protein interacts with the Cdc37/Hsp90 complex. We also investigated the interaction of P from different genotypes of lyssaviruses with Cdc37 and Hsp90. The data shown in Fig. 3G–I demonstrated that the ectopically expressed P protein from the fixed virus CVS-11 strain of genotype 1, the Australian bat lyssavirus (ABLV) of genotype 7 and the mokola virus (MOKV) of genotype 3, also bound the Cdc37/Hsp90 complex. Taken together, these data demonstrated that all lyssaviruses have a common property: the viral P protein interacts and is stabilized by the cellular Cdc37/Hsp90 complex.

### Cdc37 sustains the P protein stability with its binding domain of Hsp90

**Figure 4:**
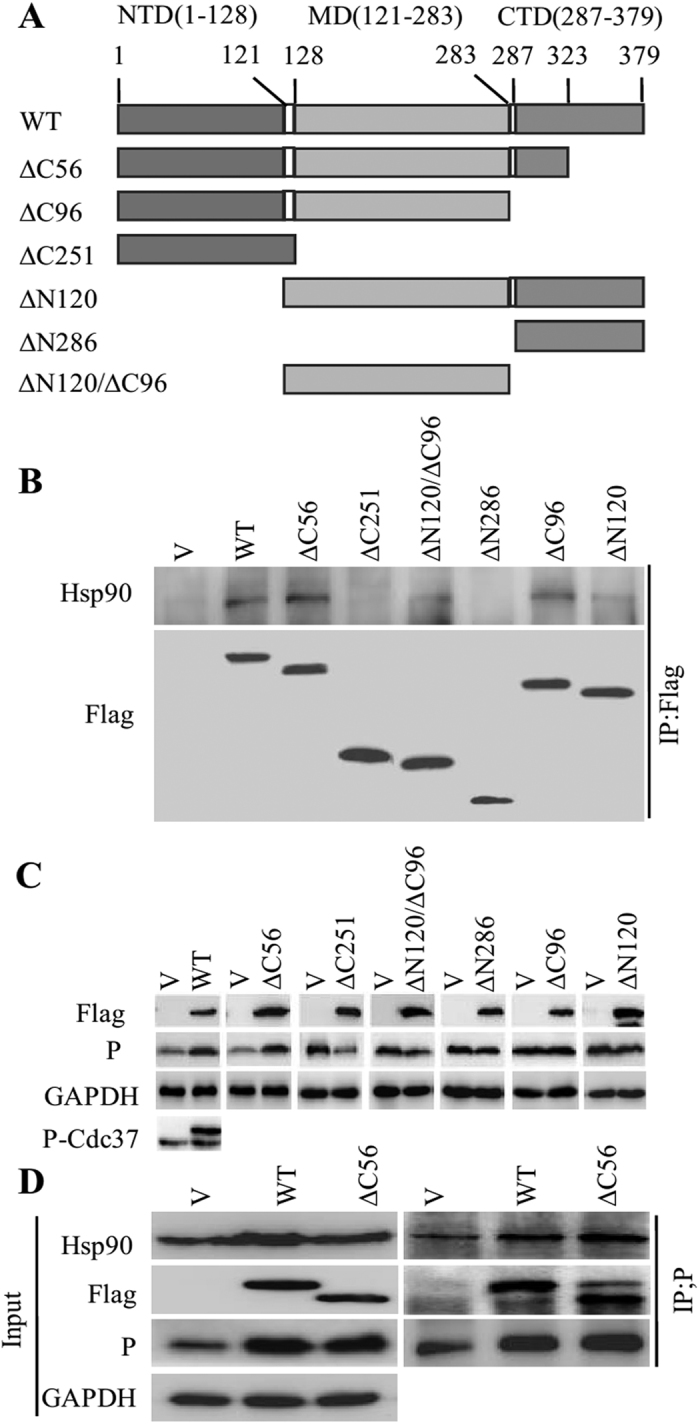
The recruitment of P to Hsp90 machinery requires the binding domain of Cdc37 for Hsp90. (**A**) Schematic diagram of Cdc37 truncation mutants. (**B**) Interaction between Hsp90 and different Cdc37 truncation mutants. Different Cdc37 truncation mutants were transfected into N2a cells for 48 h. IP was performed with mouse anti-Flag mAb and western blotting was completed using mouse anti-Flag and anti-Hsp90 mAbs. (**C**) The expression of P was determined in an immunoblotting experiment. N2a cells were transfected with empty vector (V), Flag-Cdc37 (WT) or other Cdc37 truncated mutants for 24 h, and then infected with RABV strain HEP-Flury at an MOI = 1 for 36 h. Cell lysates were probed with mouse anti-P and anti-Flag mAbs in immunoblotting experiments. The phosphorylated Cdc37 was probed with rabbit anti-Cdc37 (phosphor S13) mAb in wtCdc37 transfected N2a cells by immunoblotting. (**D**) Interaction analysis of Hsp90 and RABV P in wtCdc37 and Cdc37∆C56 transfected cells. After N2a cells were transfected with empty vector, Flag-Cdc37 and Flag-Cdc37 (∆C56) for 24 h, and then infected with RABV strain HEP-Flury at an MOI = 1 for 36 h, interaction analysis of P binding to Hsp90 was performed with mouse anti-P and rabbit anti-Hsp90 mAbs in co-immunoprecipitation and immunoblotting experiments.

Truncated Cdc37 containing its Hsp90 binding domain can stabilize kinases targets^18^,^19^,^20^. To further investigate the function of the domain of Cdc37 in P protein stability, we constructed a series of Cdc37 truncation mutants and analyzed their effects on the P protein level during RABV infection (Fig. 4A). First, the binding capacity of these mutants with Hsp90 was analyzed. A Co-IP assay showed that wild-type (WT) Cdc37, Cdc37∆C56, Cdc37∆C96, Cdc37∆N120/∆C96, Cdc37∆N120 could interact with Hsp90, while Cdc37∆C251 and Cdc37∆N286 could not (Fig. 4B), indicating aa 121–283 of Cdc37 (Cdc37∆N120/∆C96) is the critical domain for interaction with Hsp90. We then observed that P protein stability was only promoted in wtCdc37 and Cdc37∆C56 overexpressing or expressing cells, but not in Cdc37∆C96-, Cdc37∆C251-, Cdc37∆N120-, Cdc37∆N120/∆C96- and Cdc37∆N286-expressing cells (Fig. 4C), indicating that the C-terminal 56 residues of Cdc37 are not involved in P protein accumulation. Previous study have demonstrated that the increased CDK4 stability in Cdc37-overexpressing cells was due to enhanced binding to Hsp90^23^, so we speculated that the increased P stability in wtCdc37-overexpressing and Cdc37∆C56-expressing cells was also due to enhanced binding to Hsp90. Next, a Co-IP assay showed that the interaction between the P protein and Hsp90 was increased when N2a cells were transfected with wtCdc37 and Cdc37∆C56 (Fig. 4D). These data demonstrated that the N-terminal domain, middle domain and C-terminal residues aa 287–323 of Cdc37, which contain the Hsp90 binding domain, are indispensable for facilitating the stability of the P protein.

### Cdc37 sustains the stability of the P protein without binding to Hsp90

**Figure 5:**
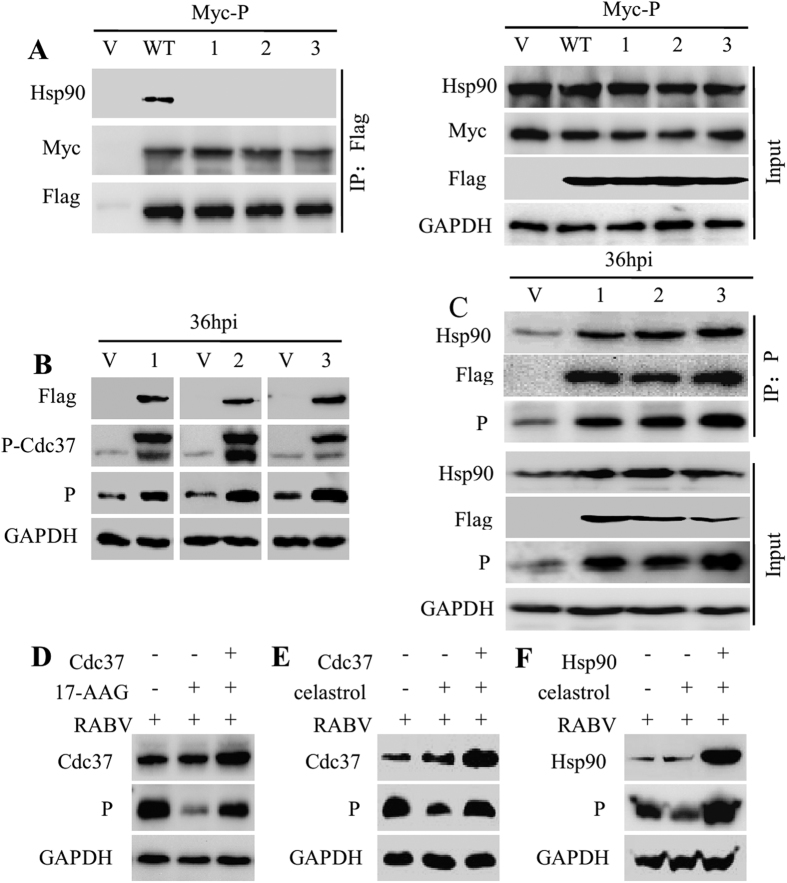
Cdc37-Hsp90 interaction is dispensable for the recruitment of P to Hsp90 machinery. (**A**) Interaction between Hsp90 and P, wtCdc37 or Cdc37 point mutants in N2a cells. Flag empty vector (V), Flag-Cdc37 (wt), Flag-Cdc37(M165A, 1), Flag-Cdc37(L206A, 2) or Flag-Cdc37(M165A/L206A, 3) were transfected into N2a cells together with Myc-P for 48 h. IP was performed with Flag antibody and immunoblotting was carried out using anti-Flag, anti-Myc, anti-Hsp90 and anti-GAPDH antibodies. (**B**) Immunoblotting of the expression of P and phosphorylated Cdc37. N2a cells were transfected with Flag empty vector, and three different Cdc37 point mutants M165A, L206A, M165A/L206A for 24 h, and then infected with RABV strain HEP-Flury at an MOI = 1 for 36 h. Cell lysates were probed with mouse anti-P and rabbit anti-Cdc37 (phosphor S13) mAbs in immunoblotting experiments. (**C**) IP analysis of cell samples described in (**B**). Protein extracts were immunoprecipitated with an anti-P antibody to detect the interaction of Hsp90, Cdc37 and P. Immunoblotting analysis was performed to detect the expression of Hsp90, P, GAPDH and three different Cdc37 mutants in the input samples. (**D**) N2a cells were transfected with PCI-neo empty vector or PCI-neo-Cdc37 for 24 h, and then incubated with RABV strain HEP-Flury at an MOI = 1 for 2 h. The infected N2a cells were treated with vehicle control or 17-AAG (500 nM) for 22 h. At 24 hpi., the harvested cell lysates were used to determine the expression level of P and Cdc37 by western blotting. (**E**) The transfected and infected N2a cells described in (**D**) were treated with vehicle control or celastrol (500 nM) for 34 h. The harvested cell lysates were probed with mouse anti-P and rabbit Cdc37 mAbs to analyze the expression of P and Cdc37 by immunoblotting. (**F**) N2a cells were transfected with PCI-neo empty vector or PCI-neo-Hsp90 for 24 h, and then incubated with RABV strain HEP-Flury at an MOI = 1 for 2 h. The infected N2a cells were incubated with vehicle control or celastrol (500 nM) for 34 h. At 36 hpi., cell lysates were harvested and the expression levels of P and Hsp90 were examined by western blotting.

Interestingly, Cdc37 can stabilize kinases without binding to Hsp90^23^. Thus, we questioned whether Cdc37 could sustain P protein stability without binding to Hsp90. To test this, based on the compared sequence of Cdc37-Hsp90 interface in Homo sapiens and Mesocricetus auratus (Supplementary Fig. 4A), we substituted the residues M165 and L206 of Cdc37 with alanine to impair the Cdc37-Hsp90 interaction, and the corresponding single/double mutants were generated. FLAG immunoprecipitation showed that the Cdc37 mutants had no detectable binding activity to Hsp90 and a similar binding capacity with the P protein compared with wtCdc37 (Fig. 5A), indicating that the mutants M165A and L206A disrupted the binding between Cdc37 and Hsp90 but not the binding between the P protein and Cdc37. The effects of these mutants on the stability of the non-kinase P protein were analyzed in RABV infected N2a cells. Notably, the P protein level was elevated when the Cdc37 mutants were expressed (Fig. 5B). Furthermore, the P protein could be immunoprecipitated from all the Cdc37 mutants-transfected N2a cells exhibiting higher P-Hsp90 association compared to that seen for cells transfected with the empty vector (Fig. 5C). In addition, and unexpectedly, expressing the Cdc37 mutants could phenocopy wtCdc37 to increase the level of P protein (Supplementary Fig. 4B). Furthermore, when Hsp90 activity was inhibited by 17-AAG or when the Cdc37 and Hsp90 interaction was disrupted by celastrol, Cdc37 overexpression could partially maintain the stability of the P protein stability (Fig. 5D,E, Supplementary Fig. 4C,D, P < 0.05, 0.01 or 0.001). However, Hsp90 overexpression could restore the P protein to a level that was higher than its original level which was originally inhibited in the presence of celastrol (Fig. 5F, Supplementary Fig. 4E, P < 0.01 or 0.001). Taken together, these results demonstrated that the Cdc37-Hsp90 interaction is dispensable for Cdc37 to chaperone the P protein during RABV infection and that Cdc37 can recruit the non-kinase P protein independent of its binding with Hsp90; however, Cdc37 cannot completely compensate for the loss of Hsp90 function.

### Cdc37 phosphorylation is not necessary for chaperoning the P protein

**Figure 6:**
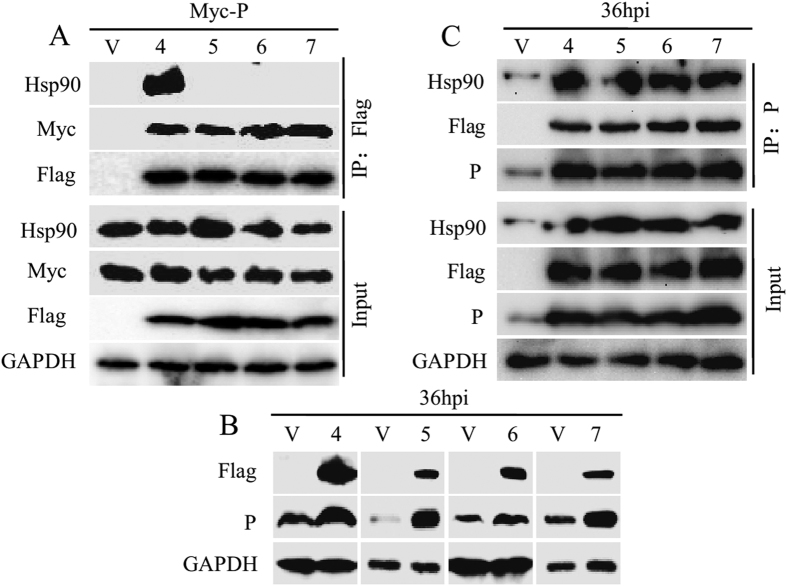
Cdc37 phosphorylation on Ser13 is not necessary for maintaining the stability of RABV P protein. (**A**) Interaction among Hsp90, P and unphosphorylated Cdc37 point mutants in N2a cells. Flag empty vector (V), Flag-Cdc37 (S13A, 4), Flag-Cdc37(M165A/S13A, 5), Flag-Cdc37(L206A/S13A, 6) or Flag-Cdc37(M165A/L206A/S13A, 7) were transfected into N2a cells together with Myc-P for 48 h. IP was performed with an anti-Flag antibody and immunoblotting was analyzed using anti-Flag, anti-Myc anti-Hsp90 and anti-GAPDH antibodies. (**B**) P expression analysis in Cdc37 point mutant transfected cells. N2a cells were transfected with (**A**)-described empty and four different Cdc37 point mutant vectors for 24 h, and then infected with RABV strain HEP-Flury at an MOI = 1 for 36 h. Immunoblotting was performed to determine P expression using an anti-P mAb. (**C**) Co-IP experiment of cell samples described in (**B**). Protein extracts were immunoprecipitated with the anti-P antibody. Immunoblotting was probed with anti-Hsp90, anti-P, anti-Flag (for detection of the four different Cdc37 point mutant in the input samples), anti-Cdc37 (for detection of the four different Cdc37 point mutant in the IP samples) and anti-GAPDH mAbs or pAbs.

Phosphorylation of Cdc37 on Ser13 is critical for kinase binding and maturation^27^,^28^; therefore, we evaluated whether it is also crucial for maintaining P protein stability. We substituted Ser13 of Cdc37 with alanine to abolish phosphorylation of Cdc37 on Ser13 (Cdc37-S13A), and verified that S13A could not be phosphorylated by western blotting using a Phospho-Cdc37 specific monoclonal antibody (mAb) (Supplementary Fig. 5A). S13A mutants Cdc37-M165A/S13A, Cdc37-L206A/S13A, Cdc37-M165A/L206A/S13A, which lacked Hsp90 binding capacity, were also generated. Surprisingly, FLAG immunoprecipitation showed that the Cdc37-S13A mutant could still interact with the P protein and Hsp90 with high efficiency (Fig. 6A, Supplementary Fig. 5B). As expected, S13A mutants lacking Hsp90 binding capacity had no detectable binding activity to Hsp90, but retained their binding with the P protein (Fig. 6A). We then tested whether these mutants were still capable of maintaining P protein stability in RABV infected N2a cells. The P protein level was elevated when the non-phosphorylatable Cdc37 mutants (M165A/S13A, L206A/S13A, M165A/L206A/S13A) were expressed (Fig. 6B), and the P protein could be immunoprecipitated from all the mutants-transfected N2a cells exhibiting more P-Hsp90 association compared to that seen for cells transfected with the empty vector (V) (Fig. 6C). Moreover, all the non-phosphorylatable Cdc37 have the similar capacity of wtCdc37 to promote the P protein stabilization (Supplementary Fig. 5C). Taken together, these results demonstrated that unphosphorylated Cdc37 could also stabilize the P protein, with or without binding to Hsp90, revealing a different mechanism of the Cdc37/Hsp90 complex chaperoning the P protein from that of chaperoning kinase.

## Discussion

Hsp90, as a chaperone, is ubiquitously expressed in mammalian cells, and is required for maturation and activation of a large number of key cellular proteins and protein complexes^8^,^9^. Systematic analyses of the interaction of Hsp90 and cellular proteins have shown that Hsp90 could associate with 7% of transcription factors, 60% of kinases and 30% of ubiquitin ligases^29^. In addition to cellular proteins, Hsp90 is also responsible for maturation and stability of numerous viral proteins^30^, and is required for viral infection by the interaction with viral polymerases, including those from hepatitis B virus and influenza virus^31^,^32^,^33^. These viral Hsp90-specific target proteins are degraded in the presence of Hsp90 inhibitors. Cdc37, as a universal kinase-specific co-chaperone, interacts with kinases, but not with transcription factors or E3 ligases, thus leading to the maturation of numerous protein kinases together with Hsp90^29^. However, up to date, although studies have reported that the chaperone Hsp90 is nearly universally required for viral protein stability^30^, only one kinase and one kinase-like protein of DNA viruses have been shown to interact with Cdc37/Hsp90 machinery. Cdc37, in concert with Hsp90, interacts with the reverse transcriptase (RT) of hepadnavirus, a kinase c-Raf like protein, to promote virus assembly and replication^34^. The Cdc37/Hsp90 complex also associates with the protein kinase of the Epstein-Barr virus (EBV PK), however, this association does not affect the stability of EBV PK^35^. In this study, we showed that the RNA virus RABV infection directly increased the expression level of cellular Hsp90 and its co-chaperone Cdc37, and in cells depleted of Cdc37 or Hsp90, viral protein expression, viral RNA synthesis and virus production were all severely inhibited. However, Cdc37 or Hsp90 overexpression only increased the expression level of the N and/or P protein, but not viral RNA synthesis or virus progeny (Fig. 1). These results indicated that Cdc37 or Hsp90 regulates N and P abundance at the protein level. Meanwhile, we further demonstrated that Cdc37 and Hsp90 positively regulated rabies virus infection by maintaining the stability of the P protein, but not the N protein, via preventing P protein clearance through autophagy (Fig. 2). Further Co-IP experiments showed that Cdc37/Hsp90 complex interacted with the P protein, not the N protein. Therefore, these data confirmed that the RABV P protein is the target of the Cdc37/Hsp90 complex. Interestingly, the RABV P protein is a transcription factor that can be phosphorylated but is not a kinase^36^. To the best of our knowledge, it is the first time to show that viral non-kinase protein can be clients for Cdc37/Hsp90 complexes. Unfortunately, we did not observe the direct interaction of the P protein with Cdc37 or Hsp90 using a GST-Pull-down assay (data not shown). Direct observation of Cdc37/Hsp90 with their clients is a known challenge, and functional studies are usually used to determine their clients candidates^29^. The possible reason might be that binary assays, such as yeast two hybrid assays or GST-Pull down assays, may not detect all Hsp90 interactions in mammalian cells due to posttranslational modifications or the absence of correct co-chaperones.

In maintaining the kinase targets of the Cdc37/Hsp90 complex, it has been shown previously that Cdc37 and Hsp90 have their own distinct and non-redundant roles^23^. Our study showed that when the activity of Hsp90 was inhibited, Cdc37 overexpression can recover the accumulation of P protein, but it cannot completely restore P protein to its original level (Fig. 5D,E). This result is consistent with previous findings suggesting that Cdc37 has its own chaperoning capacity separate from Hsp90^37^. We also demonstrated that, similarly to recruiting kinase, Cdc37 could recruit P protein to the Hsp90 machinery despite of its binding capacity with Hsp90 (Fig. 5A–C). Then we reasoned that whether the interaction between Cdc37 and Hsp90 has any additional benefit for P protein stabilization. We disrupted the interaction between Cdc37 and Hsp90 by celastrol, an allosteric inhibitor that binds to Hsp90 C-terminal domain^22^, and found that Hsp90 overexpression can restore P protein to a level that is similar or even higher than its original level. Therefore overexpression of Hsp90, but not Cdc37, can compete celastrol from binding with Hsp90, thus recover the interaction between Cdc37 and Hsp90. Collectively, these findings reveal that it is another pathway that Cdc37 without binding to Hsp90 can load P protein to Hsp90 machinery, and that Cdc37 interaction with Hsp90 might provide additional allosterical regulation of its chaperone activity although the binding capacity of Cdc37 with Hsp90 is not necessary for target stabilization.

During chaperoning of the kinase targets, phosphorylation of Cdc37 on Ser13 is critical for kinase binding and maturation^28^,^38^, and in turn, the mature kinase regulates the molecular chaperone activity of Cdc37 by phosphorylating Cdc37 on Ser13. Therefore, kinase and Cdc37 constitute a positive feedback loop to coordinate nucleotide-mediated conformational switching of Hsp90^28^. Surprisingly, in this report we found that Cdc37 not phosphorylated on Ser13 could recruit the P protein to the Hsp90 machinery, with or without binding of Cdc37 to Hsp90 (Fig. 6). This data suggested that, in contrast to the case of kinase targets, phosphorylating S13 of Cdc37 is not critical for its capacity to bind the P protein, or to coordinate P protein binding with Hsp90 to generate high affinity complexes among Hsp90, Cdc37 and P protein. However, we cannot preclude the possibility that phosphorylating S13 of Cdc37 may have additional regulatory benefits in the coordinated ATP-driven conformational switching of Hsp90 in chaperoning the P protein. This result highlighted a novel way by which Cdc37/Hsp90 chaperones its non-kinase targets. Nonetheless, given the relationship of Cdc37 with kinases and the fact that P protein can be phosphorylated, it remains possible that traditional kinase signaling pathways play an important role in the maturation of the P protein. The detailed mechanism remains to be determined.

Another interesting finding in our study is that although the N protein is not a target protein for Cdc37/Hsp90 complex, an increase/decrease of N protein expression was also observed when Cdc37 or Hsp90 expression was elevated/inhibited (Fig. 1). This observation is consistent with the notion that P protein has the role in preventing the N protein from aggregation by increasing the solubility of the N protein^39^. Indeed, the P protein is a multifunctional protein during RABV infection, acting as a non-enzymatic polymerase cofactor, dynein light chain 8 (LC8) binding and interferon antagonist^40^,^41^,^42^,^43^,^44^,^45^. The N protein interacts with Hsp70^5^, thus Hsp70-N protein complexes and Cdc37-Hsp90-P protein complexes may interact with each other through an N-P interaction. Further exploration of this mechanism might provide insights into how the virus life cycle is controlled.

**Figure 7:**
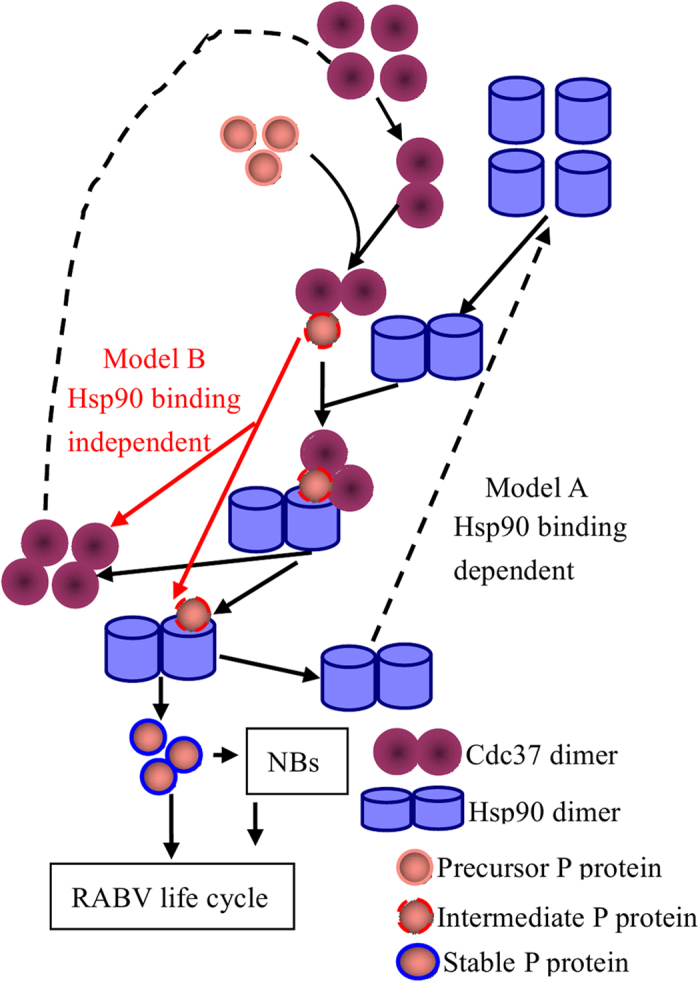
The mechanistic model of Cdc37/Hsp90’s stabilization of the non-kinase target P. Two major models were constructed for the role of Cdc37/Hsp90 machinery in stabilizing the RABV P protein. Model A indicates that Cdc37 sustains the stability of the RABV P protein by binding to Hsp90. In model B, Cdc37 sustains the stability of the RABV P protein without binding to Hsp90. In both cases, phosphorylation of Cdc37 on Ser13 is not required.

In summary, we have demonstrated that Cdc37 acts as a bridge to direct Hsp90 to target a set of novel non-kinase targets, the phosphorylatable, transcription factor viral P proteins of all the genotypes of lyssaviruses. Although Cdc37 can load immature P proteins onto Hsp90, either with or without binding to Hsp90, the interaction between Cdc37 and Hsp90 appears to provide additional allosterical regulation of its chaperone activity. Notably, both phosphorylated and non-phosphorylated Cdc37 could facilitate Hsp90-mediated P protein maturation. This work complements and extends the current model for the mechanism of Cdc37 action in coordinating with Hsp90 in chaperoning its targets (Fig. 7).

## Methods

### Cell culture and virus infection

Mouse neuroblastoma N2a cells were provided by Professor Xiaofeng Guo from South China Agricultural University, Guangzhou, China and were maintained in Dulbecco’s modified Eagle’s medium supplemented with 5% heat-activated fetal calf serum (Gibco/Invitrogen, Carlsbad, CA, USA). The RABV strains HEP-Flury and CVS-11 were stored in our laboratory. During the experiment, N2a cells were infected with RABV at the indicated multiplicities of infection (MOI).

### Antibodies and Reagents

Mouse monoclonal antibodies (mAbs) against RABV N or P were prepared in our laboratory^46^. The rabbit polyclonal antibody (pAb) against GAPDH was purchased from Hangzhou Good Here Biotechnology Co. Ltd (Hangzhou, China). Rabbit anti-ß-actin and anti-Myc pAbs were obtained from Hangzhou Huaan Biotechnology Co. Ltd (Hangzhou, China). Rabbit anti-Hsp90AA1, anti-Cdc37 (phospho S13) and anti-Cdc2 mAbs were purchased from Abcam (Cambridge, MA, USA). Rabbit anti-LC3B, anti-Cdc37 and anti-IKKa mAbs were purchased from Cell Signaling Technology (Beverly, MA, USA). Mouse anti-Flag (clone M2) mAb was purchased from Sigma-Aldrich (St. Louis, MO, USA). Alexa Fluor 546-conjugated donkey anti-mouse IgG and Alexa Fluor 647-conjugated goat anti-rabbit IgG were purchased from Invitrogen Life Technologies (Carlsbad, CA, USA). The proteasome inhibitor MG-132 and the de novo protein synthesis inhibitor cycloheximide (CHX) were purchased from Beyotime Biotechnology (Shanghai, China). The Hsp90 inhibitor 17-(Allylamino)-17-demethoxygeldanamycin (17-AAG), the Hsp90 allosteric inhibitor celastrol and the autophagy inhibitor wortmannin were purchased from Sigma.

### Treatment of cells with inhibitors

RABV-infected cells were treated with different concentrations of 17-AAG, celastrol, MG-132, wortmannin or CHX for the indicated times before the cells were harvested. DMSO, as the vehicle of the above-mentioned inhibitors, was used as a non-treatment control.

### Plasmids constructs and transfection

The specific primers for constructs generated in this study are listed in Supplemental Table 1. The full-length Hsp90 gene was amplified from the cDNA of N2a cells and cloned into the plasmid pCI-neo (Promega, Madison, WI, USA). The wtCdc37 gene was amplified from N2a cells cDNA and cloned into pCMV-N-Flag (Clontech, Palo Alto, CA, USA) or pCI-neo. The full-length P gene was amplified from the cDNA of HEP-Flury (Accession: AB085828.1) and cloned into pCMV-N-Flag and the pCMV-N-Myc (Clontech). The cDNAs of the full-length P genes of RABV strains CVS-11 (Accession: GQ918139.1), RV4 (MOKV; Accession: KF155005.1) and RV634 (ABLV; Accession: AF418014.1) were obtained from Prof. Changchun Tu (Institute of Military Veterinary Science, the People’s Liberation Army, Changchun, China) and cloned separately into pCMV-N-Flag. To construct the recombinant plasmids pSG5-P and pSG5-N, the cDNA segments of the viral genes P and N were cloned into plasmid pSG5 (Agilent technologies, Santa Clara, CA, USA), separately. The truncated fragments of the Cdc37 gene (Fig. 4A) were separately inserted into pCMV-N-Flag. To generate the single and double point mutants of Cdc37 gene a Site-directed Gene Mutagenesis Kit (Beyotime Biotechnology) was used with the corresponding specific primers and cloned into pCMV-N-Flag. Transfection of supercoiled plasmid DNA was performed with ExFect^TM^ Transfection Reagent (Vazyme Biotechnology, Nanjing, China).

### Western blotting

Western blotting was performed as previously described^47^. Briefly, cells were lysed in lysis buffer after infection or other treatments for the indicated times. Whole cellular proteins were harvested by lysing cells in SDS lysis buffer (50 mM Tris-Cl pH 7.4, 1% SDS, 1% Triton X-100, 1 mM PMSF) on ice for 5 min, resuspended in 4 × SDS-PAGE loading buffer (Takara, Dalian, China) and boiled for 10 min. After centrifugation, the soluble cell lysates were separated on 12% SDS-PAGE gels, transferred to nitrocellulose membranes, and subjected to immunoblot analysis. Membranes was blocked with 5% nonfat dry milk in PBS for 1 h at 37 °C, and incubated with the indicated primary antibodies overnight at 4 °C. The membrane was then incubated with an appropriate secondary antibody conjugated to horseradish peroxidase (HRP) (KPL, Gaithersburg, MD, USA) for 1 h at 37 °C. The blots were developed with SuperSignal West Femto maximum sensitivity substrate (Thermo Fisher Scientific, Rockford IL, USA) according to the manufacturer’s protocol. Images were captured using optimal auto-exposure settings on a chemiluminescent imaging system (Cell Biosciences, Santa Clara, CA, USA) and quantified using ImageJ software (National Institutes of Health, Bethesda, Maryland, USA).

### Co-immunoprecipitation

Co-immunoprecipitation was performed as previously described, with some modifications^48^. Briefly, the infected or transfected N2a cells were washed twice with cold PBS and lysed in cell lysis buffer for Western and IP (Beyotime Biotechnology) with 1 mM phenylmethylsulfonyl fluoride (PMSF) protease inhibitor (Beyotime Biotechnology) overnight at 4 °C. Cell lysates were centrifuged at 12,000 × g for 10 min at 4 °C to remove insoluble fractions. The soluble fractions were pretreated with protein A/G agarose beads (Santa Cruz Biotechnology, Santa Cruz, CA, USA) for 30 min at 4 °C. Pretreated supernatants were incubated with immunoprecipitation (IP) antibody overnight at 4 °C. Fresh protein A/G agarose was then added at 4 °C for 6 h before washing with PBS. The bound proteins were eluted by boiling in 4 × SDS-PAGE loading buffer and subjected to western blotting analysis.

### Quantitative real-time reverse transcription-PCR

Quantitative analysis of the mRNA transcripts and anti-genomic RNA were conducted according to a previous publication, with some modifications^6^. Briefly, total RNA was extracted from cells using the TRIZOL Reagent (Invitrogen), according to the manufacturer’s instructions. The total RNA was reverse transcribed into cDNAs using a RevertAid first strand cDNA synthesis kit (Fermentas, Ontario, Canada). The cDNA amplification was performed with specific primers: forward 5′-AAGGAGTTGAATGACAGGGTGCCA-3′ and reverse 5′-ACT TGGGATGGTTCGAAAGGAGGA-3′ for the RABV anti-genome (115 bp in length), forward 5′-AGCAGCAATGCAGTTCTTTGAGGG-3′ and reverse 5′-TTGTCAGTTCCATGCCTCCTGT CA-3′ for the RABV N gene (164 bp in length), and forward 5′-TCAACAGCAACTCCCACTCTTCCA-3′ and reverse 5′-ACCCTGTTGCTGTAGCCGTATTCA-3′ for GAPDH (92 bp in length).

The qRT-PCR was performed using 1 × SYBR premix EX-Taq (Perfect real time, Takara, Dalian, China) and an ABI7500 real-time PCR system (Applied Biosystems, Foster City, CA, USA). PCR conditions were as follows: 50 °C for 2 min; 95 °C for 30 s; and 40 cycles of 95 °C for 5 s and 60 °C for 34 s. A melting curve was obtained following PCR procedures. Quantitative analysis was performed using the 7500 software (version 2.0.6) with a relative quantification method (∆∆Ct) to analyze the changes in the levels of viral N mRNA and anti-genomic RNA.

### Confocal microscopy

N2a cells were grown to 80% confluence on glass cover slips overnight and infected with HEP-Flury at an MOI = 1. At 24 hpi, cells were fixed with cold acetone-methanol (1/1) for 20 min at −20 °C. The fixed cells were double stained using mouse anti-P mAb plus rabbit anti-Hsp90AA1 mAb or rabbit anti-Cdc37 mAb at 37 °C for 1.5 h, washed with PBS and incubated with Alexa Fluor 546-conjugated donkey anti-mouse IgG and Alexa Fluor 647-conjugated goat anti-rabbit IgG for 1 h at 37 °C. The cells were then stained with 4′,6-diamidino-2-phenylindole (DAPI). Confocal images were obtained using an LSM 700 laser scanning confocal microscope (Zeiss, Oberkochen, Germany).

### shRNA constructs and transfection

Hsp90 or Cdc37 knockdown in N2a cells was performed using the vector-based shRNA approach. pcDNA6.2-GW/EmGFP-miR-based shRNA for Hsp90 knockdown (NM_010480.5; target sequence ATCAATCTTTCCCAGCAAA) and the scrambled shRNA vector (target sequence GTCTCCACGCAGTACATTT) were purchased from Invitrogen; pGPU6/GFP/Neo-based shRNA for knockdown of Cdc37 (NM_016742.4; target sequence GCAAGAGCATGGTCAATACCA) and the scrambled shRNA vector (target sequence GTTCTCCGAACGTGTCACGT) were purchased from GenePharma (Shanghai, China). N2a cells at 80% confluence were transfected with shRNA constructs using ExFect^TM^ Transfection Reagent (Vazyme Biotechnology), according to the manufacturer’s instructions. After 24 h of growth at 37 °C, cells were infected with the HEP-flury at an MOI = 1 for the indicated times.

### Cell viability

The viability of N2a cells was determined using the cell Counting Kit-8 (CCK-8) assay (Beyotime Biotechnology). N2a cells were plated at a density of 2 × 10^4 ^ cells/well in 96-well plates in 100 μl Dulbecco’s modified Eagle’s medium supplemented with 5% heat-activated fetal calf serum and antibiotics, and then allowed to grow for 12 h before treatment. Cells were treated by adding 100 μl of different concentrations of the indicated drugs (17-AAG or celastrol). After treatment with 17-AAG for 24 h or with celastrol for 36 h, 20 μl CCK-8 solution was added to the cells, which were incubated at 37 °C with 5% CO_2_ for 2 h, after which the absorbance at 450 nM was measured.

### Statistical analysis

The Student’s test was used to measure the statistically significant differences between groups. A *P* value of less than 0.05 was considered statistically significant.

## Additional Information

**How to cite this article**: Xu, Y. *et al.* The co-chaperone Cdc37 regulates the rabies virus phosphoprotein stability by targeting to Hsp90AA1 machinery. *Sci. Rep.* **6**, 27123; doi: 10.1038/srep27123 (2016).

## Supplementary Material

Supplementary Information
