# Supplementary material for: The co-chaperone Cdc37 regulates the rabies virus phosphoprotein stability by targeting to Hsp90AA1 machinery
Source: Sci Rep. 2016 Jun 2;6:27123. doi: 10.1038/srep27123 (PMC4890047; doi:10.1038/srep27123)
Supplement: Supplementary Information [file srep27123-s1.doc]

Supplementary Information for Scientific Reports

**The co-chaperone Cdc37 regulates the rabies virus phosphoprotein stability by targeting to Hsp90AA1 machinery**

Yunbin Xu^1,3^, Fei Liu^2^, Juan Liu^1,3^, Dandan Wang^1^, Yan Yan^1,3^, Senlin Ji^2^, Jie Zan^1,3^, Jiyong Zhou^1,2,3^

1 Key Laboratory of Animal Virology of Ministry of Agriculture, Zhejiang University, Hangzhou 310058, PR China

2 College of Veterinary Medicine, Nanjing Agricultural University, Nanjing 210095, PR China

3 Collaborative Innovation Center and State Key Laboratory for Diagnosis and Treatment of Infectious Diseases, First Affiliated Hospital, Zhejiang University, Hangzhou 310003, PR China

*Correspondence: Jiyong Zhou, Key Laboratory of Animal Virology of Ministry of Agriculture, Zhejiang University, 866 Yuhangtang Road, Hangzhou 310058, P. R. China. Phone: +86-571-8898-2698. Fax: +86-571-8898-2218. Email: [jyzhou@zju.edu.cn](mailto:jyzhou@zju.edu.cn).

**
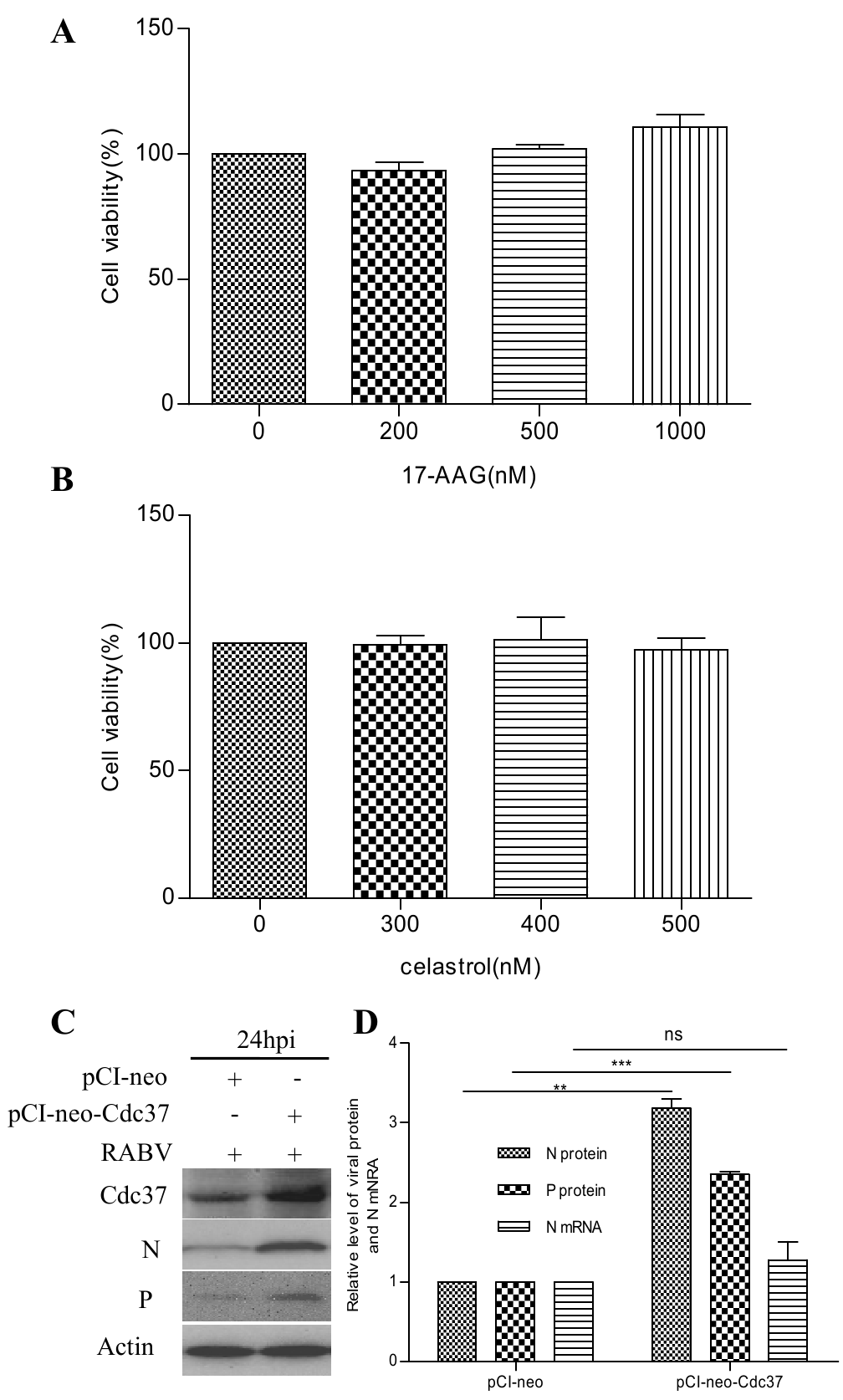
**

**Supplemental Figure 1. Cell viability and Cdc37 overexpression.** (A and B) Cell viability of N2a cells treated with different concentrations of 17-AAG or celastrol. (C) N2a cells were transfected with PCI-neo-Cdc37 for 24 h, and then infected with HEP-Flury at an MOI=1 for the indicated times. Cell extracts were analyzed by immunoblotting using anti-Cdc37, anti-N, anti-P and anti-Actin antibodies. (D) Quantitative analysis of viral N mRNA, proteins N and P, described in C. Error bars: Mean ± SDs of three independent experiments. ^ns^ *P* > 0.05, ^**^ *P* < 0.01, ^***^ *P* < 0.001.


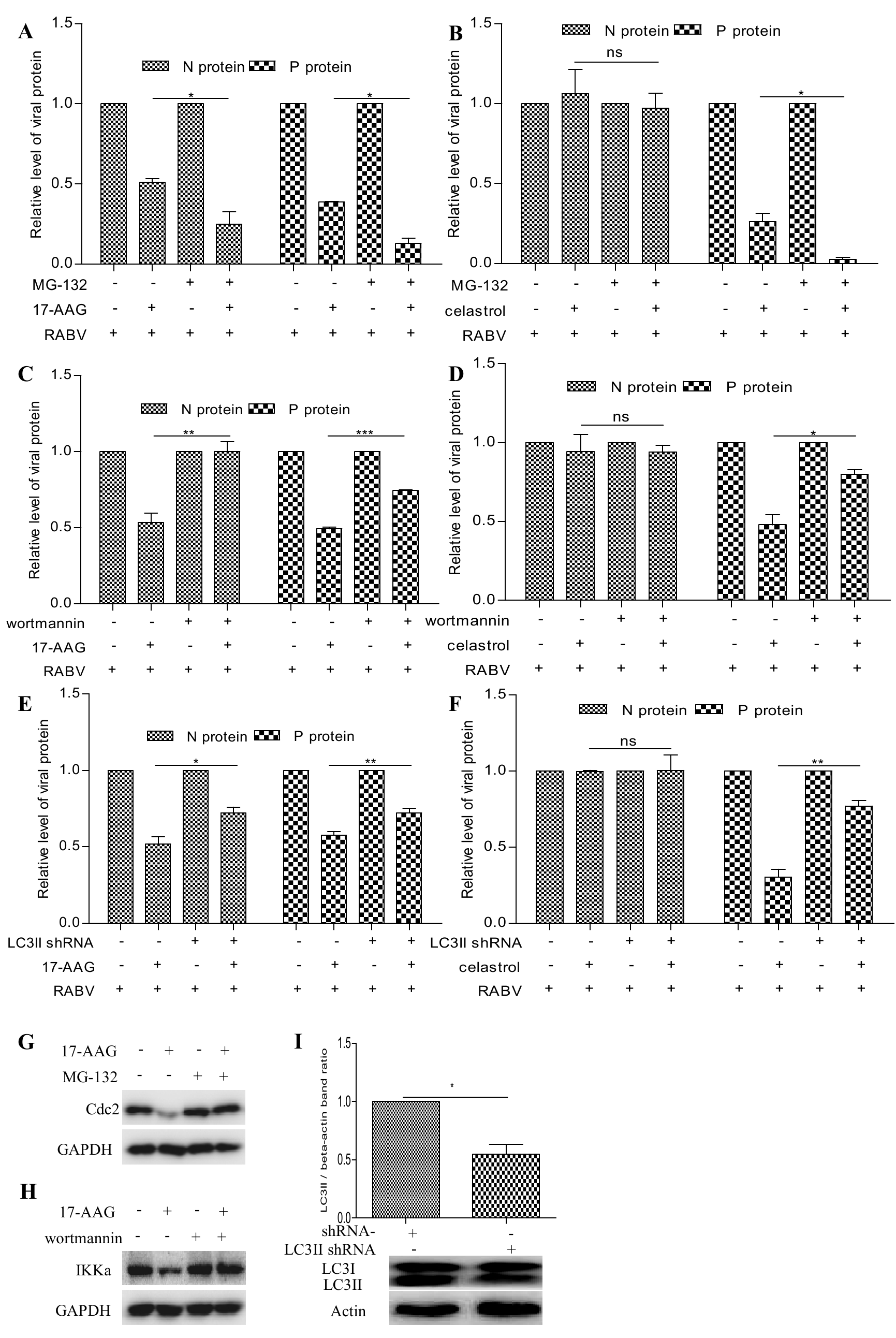


**Supplemental Figure 2. (A-F) Quantitative analysis of N and P protein levels described in Figure 2E-2J. (G) Western blotting analysis of the effect of MG-132 on Cdc2 degradation in the presence of 17-AAG. (H) Western blotting analysis of the effect of wortmannin on IKKa degradation in the presence of 17-AAG. (I) Quantitative analysis of LC3 II level in negative control shRNA (shRNA(-)) and LC3II shRNA-transfected N2a cells after 48h transfection.** Error bars: Mean ± SDs of three independent experiments. ^ns^ *P* > 0.05, ^*^ *P* < 0.05, ^**^ *P* < 0.01, ^***^ *P* < 0.001.


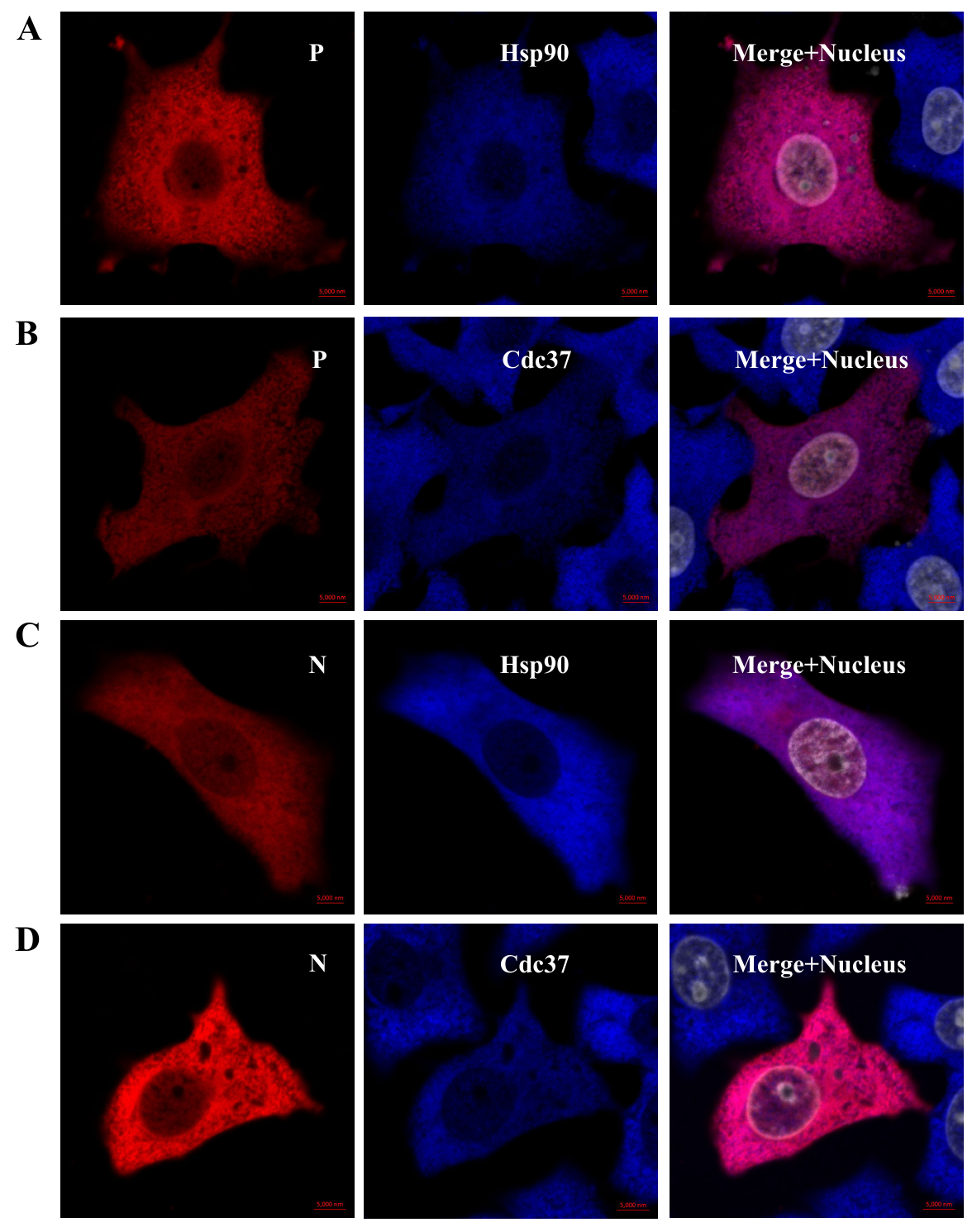


**Supplemental Figure 3. (A-D) Subcellular distribution of Cdc37 and Hsp90 in RABV N or P transfected N2a cells by confocal microscopy.**

**
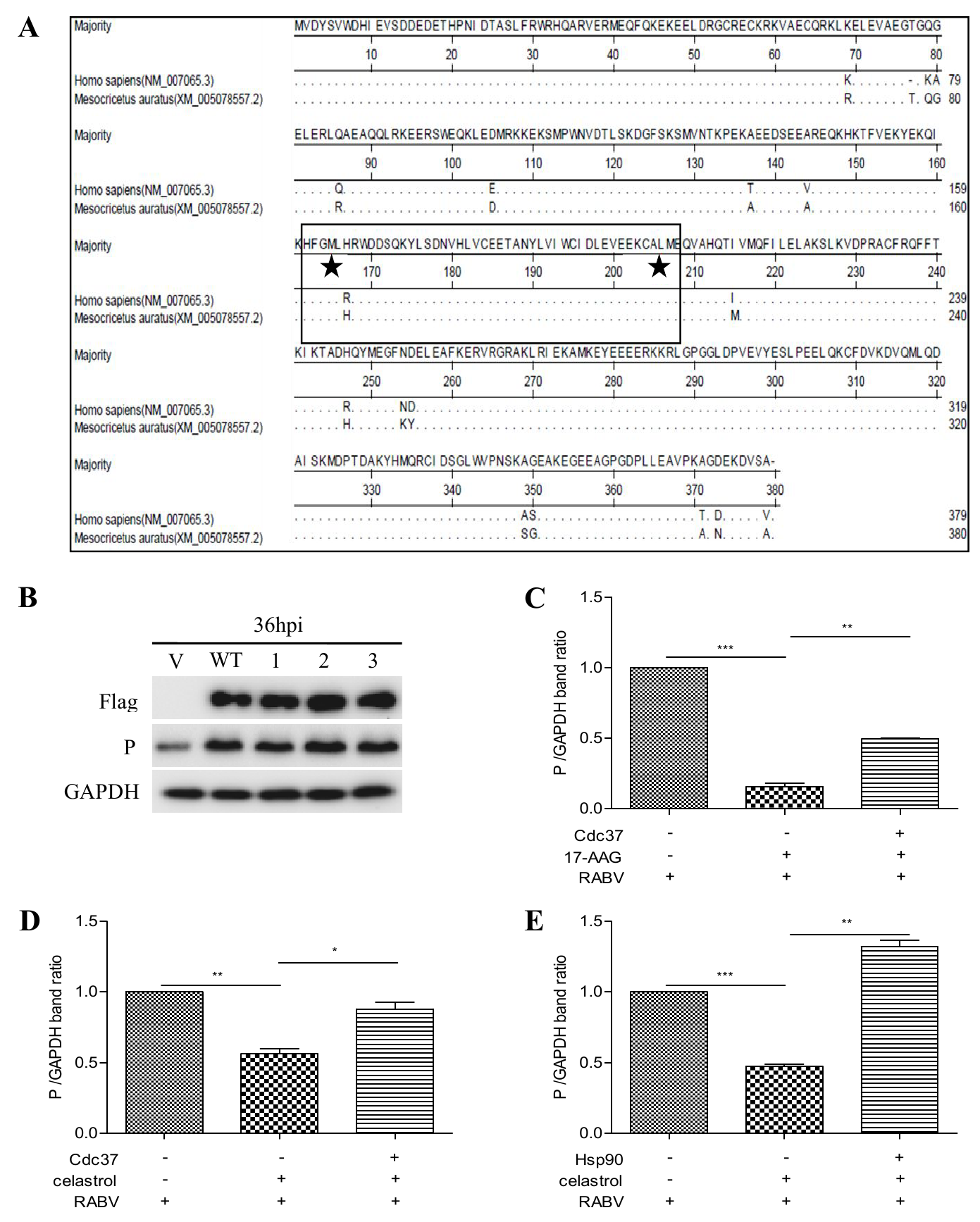
**

**Supplemental Figure 4. (A) The alignment of Cdc37 amino acid sequence between Homo sapiens and Mesocricetus auratus.**□, the Hsp90 binding domain of Cdc37.★, the binding residues. (B) **P expression analysis in wtCdc37 and Cdc37 point mutant transfected cells.** N2a cells were transfected with Flag empty vector (V), Flag-Cdc37 (wt), Flag-Cdc37(M165A, 1), Flag-Cdc37(L206A, 2) or Flag-Cdc37(M165A/L206A, 3) for 24 h, and then infected with RABV strain HEP-Flury at an MOI=1 for 36 h. Immunoblotting was performed to determine P expression using an anti-P mAb. (C-E) **Quantitative analysis of P protein levels described in Figure 5D-5F.** Error bars: Mean ± SDs of three independent experiments. ^*^ *P* < 0.05, ^**^ *P* < 0.01, ^***^ *P* < 0.001.


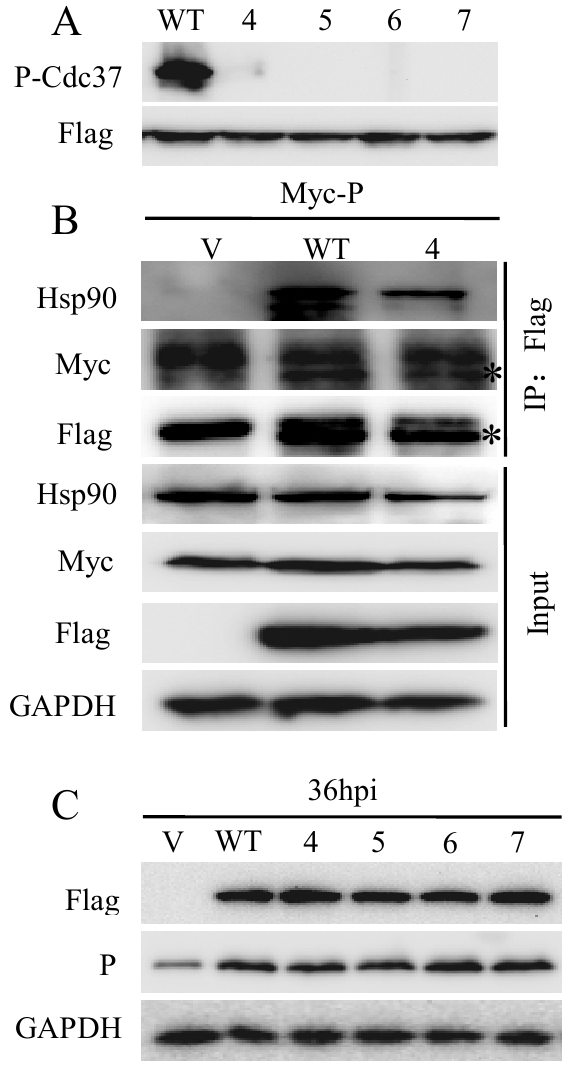


**Supplemental Figure 5. Identification of Cdc37 phosphorylated mutants.** (A) Identification of Ser13 phosphorylation. The plasmids Flag-Cdc37 (wt), Flag-Cdc37 (S13A, 4), Flag-Cdc37 (M165A/S13A, 5), Flag-Cdc37 (L206A/S13A, 6) or Flag-Cdc37 (M165A/L206A/S13A, 7) were transfected separately into N2a cells for 48 h. The total cell lysates were resolved by SDS-PAGE and analyzed by immunoblotting using anti-Flag and anti-Cdc37 (phosphor S13) mAbs. (B) Flag empty vector (V), Flag-Cdc37 (WT) or Flag-Cdc37 (S13A,4) together with Myc-P were co-transfected into N2a cells for 48 h. Protein extracts were immunoprecipitated with an anti-Flag antibody and the immune complexes were immunoblotted with anti-Flag, anti-Myc and anti-Hsp90 mAbs. * indicates the specific protein band; the band above this specific band is the IgG(H) band. (C) Immunoblotting of the expression of P. N2a cells were transfected with Flag empty vector (V), Flag-Cdc37 (wt), Flag-Cdc37 (S13A, 4), Flag-Cdc37(M165A/S13A, 5), Flag-Cdc37(L206A/S13A, 6) or Flag-Cdc37(M165A/L206A/S13A, 7) for 24 h, and then infected with RABV strain HEP-Flury at an MOI=1 for 36 h. Cell lysates were probed with mouse anti-P mAb in immunoblotting experiments.

**Supplemental Table 1.** Primers used for plasmids construction

| Plasmids | Sense primer(5’-3’) | Anti-sense primer(5’-3’) |
| --- | --- | --- |
| PCI-neo-Hsp90 | CCGCTCGAGATGCCTGAGGAAACCCAGAC(*Xho* I) | ACGCGTCGACTTAGTCTACTTCTTCCATGCGTG(*Sal* I) |
| PCMV-N-Flag-Cdc37 | CGGAATTCCGATGGTGGACTACAGCGTTTG(*Eco*RI) | AAGGAAAAAAGCGGCCGCTCACGCACTGACGTCTTTCTC(*Not* I) |
| PCI-neo-Cdc37 | CCGGAATTCATGGTGGACTACAGCGTTTG(*Eco*RI) | GCTCTAGATCACGCACTGACGTCTTTCT(*xba* I) |
| PCMV-N-Flag-P(HEP-Flury) | CGGAATTCCGATGAGCAAGATCTTTGTTAATC(*Eco*RI) | CCCTCGAGG TTAGCATGATGTGTAGCG(*Xho* I) |
| PCMV-N-Flag-P(CVS-11) | CGGAATTCCGATGAGCAAGATCTTTGTTAAT(*Eco*RI) | CCCTCGAGGTTAGCAGGATGTATAGCGATT(*Xho* I) |
| PCMV-N-Flag-P(ABLV) | CGGAATTCCGATGAGCAAGATCTTTGTCAATCC(*Eco*RI) | CCCTCGAGGTCAACATGACATRTAACGGTTCA(*Xho* I) |
| PCMV-N-Flag-P(MOKV) | CGGAATTCCGATGAGCAAGGACCTTGTGC(*Eco*RI) | CCCTCGAGGCTATTCTGCATCCTCAAGC(*Xho* I) |
| PCMV-N-Myc-P(HEP-Flury) | CGGAATTCCGATGAGCAAGATCTTTGTTAATCCGAG(*Eco*RI) | ACGCGTCGACGTTTAGCATGATGTGTAGCGATCCAAGT(*Sal* I) |
| PCMV-N-Flag-Cdc37(∆C56) | CGGAATTCCGATGGTGGACTACAGCGTTTG(*Eco*RI) | AAGGAAAAAAGCGGCCGCTCACTAGCTGATGGCGTCTTGC(*Not* I) |
| PCMV-N-Flag-Cdc37(∆C251) | CGGAATTCCGATGGTGGACTACAGCGTTTG(*Eco*RI) | AAGGAAAAAAGCGGCCGCTCAGCTCTTGCTGAAGCCAT(*Not* I) |
| PCMV-N-Flag-Cdc37(∆N120/∆C96) | CGGAATTCCGATGAGCAAGGATGGCTTCAG(*Eco*RI) | AAGGAAAAAAGCGGCCGCTCACTCCTCCTCCTCGTACT(*Not* I) |
| PCMV-N-Flag-Cdc37(∆N286) | CGGAATTCCGATGAGGCTGGGCCCTGGT(*Eco*RI) | AAGGAAAAAAGCGGCCGCTCACGCACTGACGTCTTTCT(*Not* I) |
| PCMV-N-Flag-Cdc37(∆C96) | CGGAATTCCGATGGTGGACTACAGCGTTTGG(*Eco*RI) | AAGGAAAAAAGCGGCCGCTCACTCCTCCTCCTCGTACTC(*Not* I) |
| PCMV-N-Flag-Cdc37(∆N120) | CGGAATTCCGATGAGCAAGGATGGCTTCAG(*Eco*RI) | AAGGAAAAAAGCGGCCGCTCACGCACTGACGTCTTTCTC(*Not* I) |
| PCMV-N-Flag-Cdc37(M165A) | gatcaagcattttggcgcgctccaccgctgggatg | catcccagcggtggagcgcgccaaaatgcttgatc |
| PCMV-N-Flag-Cdc37(L206A) | gaggagaaatgtgcggcgatggagcaggtggcgc | gcgccacctgctccatcgccgcacatttctcctc |
| PCMV-N-Flag-Cdc37(S13A) | gatcacatcgaggtggcggacgacgaggacg | cgtcctcgtcgtccgccacctcgatgtgatc |
| pSG5-N | CCGGAATTCATGGATGCCGACAAGATTG(*Eco*RI) | CGCGGATCCTTATGAGTCACTCGAATACG(*Bam*HI) |
| pSG5-P | CCGGAATTCATGAGCAAGATCTTTGTTAATC(*Eco*RI) | CGCGGATCC TTAGCATGATGTGTAGCG(*Bam*HI) |
